# Constructing socioeconomic index (SEI) in predicting mental health in young adults

**DOI:** 10.1192/j.eurpsy.2021.337

**Published:** 2021-08-13

**Authors:** C.S. Wong, W.C. Tang, W. Chang, C.L. Hui, S.K. Chan, E.H. Lee, E.Y. Chen

**Affiliations:** Department Of Psychiatry, The University of Hong Kong, HK, Hong Kong PRC

**Keywords:** Socioeconomic index, mental health, Young adults

## Abstract

**Introduction:**

Socioeconomic status (SES) are well known to be associated with mental health. Previous studies are often restricted by the use of individual SES indicators, while contextual measures aggregating multiple dimensions would present a better picture of SES in multivariate context.

**Objectives:**

The present study aims to construct the socioeconomic index (SEI) by integrating significant socioeconomic factors in predicting mental health of young adults in Hong Kong.

**Methods:**

Data were drawn from the Hong Kong Youth Epidemiological Study of Mental Health (HKYES), a population-based psychiatric study of young people in Hong Kong. The present study exacted data of 1,164 participants who had completed baseline interviews between April 2019 to August 2020. Socioeconomic characteristics including age, gender, education years, income, expenditure, home ownership, housing type, household crowdedness and parental occupation were collected. Data were checked for the assumptions for normality, linearity and homoscedasticity before the standardized SEI were derived using Principal Component Analysis (PCA). Logistic regression analyses were performed to further examine the association between SEI and mental health outcomes.

**Results:**

Our results identified five significant socioeconomic factors (education years, personal income, home ownership, housing type and household crowdedness) which together explained 67.7% of the total variation. SEI was associated with depression (OR=0.671, p=.003) and anxiety (OR=0.667, p=.015) after adjusting for potential confounders.

**Conclusions:**

The PCA-generated SEI took account of the multiple dimensions of SES in younger adults including education, income, expenditure and housing. The indices would provide meaningful contextual information of SES across geographical areas or different groups of interest.

**Disclosure:**

No significant relationships.

